# Medical practitioners’ knowledge and awareness of multiple myeloma at public hospitals, Gauteng, South Africa

**DOI:** 10.4102/safp.v65i1.5644

**Published:** 2023-06-27

**Authors:** Andiswa M. Pooe, Abegail N. Dlova, Sam T. Ntuli

**Affiliations:** 1 Department of Haematology, Faculty of Health Sciences, Sefako Makgatho University, Pretoria, South Africa; 2 Department of Statistics, Faculty of Health Sciences, Sefako Makgatho University, Pretoria, South Africa

**Keywords:** multiple myeloma, awareness, knowledge, Gauteng Province, South Africa

## Abstract

**Background:**

Multiple myeloma (MM) is a plasma cell malignancy associated with morbidity and mortality worldwide, and most patients are referred for specialist care very late with complications. The low index of suspicion among medical practitioners is among the reasons for the delay in MM diagnosis and management. This study aimed to determine the level of awareness and knowledge of MM among medical practitioners working in public hospitals of Tshwane Municipality, Gauteng Province, South Africa.

**Methods:**

A cross-sectional descriptive study on 74 doctors working in three district, one regional and one central hospital using a convenience sampling.

**Results:**

Seventy-four medical practitioners participated in this study. Their median age was 37 years with an interquartile range of 43–30 years. The majority (85%) of the respondents were aware of MM, while 74% were knowledgeable regarding MM presentations and diagnostic investigations.

**Conclusion:**

The findings highlighted a high level of awareness and knowledge of MM among the study population, but almost all of the participants requested an educational information brochure on MM.

**Contribution:**

Medical practitioners have a high level of awareness of multiple myeloma; however, there is a discrepancy between this level of awareness and the delayed presentation of patients at the public hospitals. As primary healthcare in South Africa is nurse-driven, the study indicates that not all primary healthcare providers may be aware of this disease. Future awareness campaigns should target other primary healthcare providers, including nurses and private general practitioners.

## Introduction

Multiple myeloma (MM) is a haematological malignancy characterised by the accumulation of malignant plasma cells in the bone marrow resulting in anaemia and other cytopenias, bone lesions, hypercalcaemia, renal insufficiency and monoclonal gammopathy.^1^ The incidence of MM in South Africa is 4.34, whereas in the rest of the world, it varies between 0.54 and 5.3 per 1 001 000 population.^1,2^ Multiple myeloma comprises about 1% of all malignant tumours, accounts for 10% – 15% of all haematological malignancies^2^ and is a major cause of morbidity and mortality in both developed and developing countries.^3,4^ The condition mainly affects elderly people aged 65 years and older^5,6,7,8^ and, in most cases, develops as an asymptomatic premalignant condition known as a monoclonal gammopathy of undetermined significance (MGUS).^9^ The diagnosis of MM includes the presence of this monoclonal protein in serum or urine, bone marrow clonal plasma cells and related organ or tissue impairment as evidenced by hypercalcaemia, renal insufficiency, anaemia and/or bone lesions.^2,9^ As the diagnostic criteria and management for MM have changed dramatically over the last few years, one needs to have a high index of suspicion to make the diagnosis because of its nonspecific clinical features.^10^

Globally, MM remains a major public health concern; the number of cases has increased 2.36 times from 65 940 in 1990 to 155 688 in 2019, while the mortality rate increased 2.19-fold from 51 862 to 113 474.^4^ This could be indicative of an increasing global burden for MM as the world aging population increases. In South Africa (SA), the National Cancer Registry indicates that of the 14 616 haematological malignancies reported between 2000 and 2006, MM is diagnosed in about 1543 (10.6%) of the cases.^11^ In the Eastern Cape province of SA, 3603 incident cases of haematological malignancies were identified between 2004 and 2013 and MM accounted for 465 (13%) of the cases.^12^

At Steve Biko Academic Hospital, a retrospective study was conducted between May 2005 and September 2008; MM was reported in 6.7% (*n* = 39) of 582 patients in which protein electrophoresis was performed.^13^ An earlier study conducted among 145 patients diagnosed with haematological malignancies at Dr George Mukhari Academic Hospital (DGMAH) between January 1998 and December 1998 found that MM accounted for 26% of the cases and most of these patients were referred and diagnosed late (unpublished findings).^14^

Various efforts have been made to develop standard management protocols for MM;^15,16,17^ however, the diagnosis and management of MM patients remain a challenge.^18^ Several factors may have contributed to the challenges, including the late presentation of patients to a healthcare facility,^13,19,20^ the inadequacy of diagnostic facilities^20^ and MM patients experiencing multiple consultations in the primary care before being referred to the tertiary facility.^20,21,22^ Visser et al. in their study at Steve Biko Academic Hospital reported that the majority of the MM cases were diagnosed at a very late stage of the disease and concluded that this could be related to a low index of suspicion among referring medical practitioners.^13^ This finding is supported by many studies.^21,22,23,24^

Studies have been conducted in developed countries that assessed the level of awareness and knowledge of MM practices among haematology healthcare professionals^25^ and general practitioners,^26^ but these studies were on MGUS and highlighted a lack of awareness and understanding with mean scores of 2.1 and a standard deviation of ± 1.09. The studies were done using an online questionnaire. In sub-Saharan African countries, there is a paucity of information on the awareness and knowledge of MM among medical practitioners, but in Kenya^27^ and Nigeria,^28^ researchers reported very low awareness of MM among practitioners without conducting formal research. This was based on the observed increase in patient enrolment to the MM diagnosis and management program after the training of medical students and healthcare practitioners. In our institution, the number of patients with MM is on the increase. These patients present late, and 22% of them were aged less than 40 years, which might be one of the reasons for a low index of suspicion among referring medical practitioners.^14^ Multiple myeloma is known to be preceded by pre-existing MGUS.^29,30^ Despite this, the level of awareness and knowledge of MM practices among medical practitioners remains unresolved worldwide. Therefore, this study aimed to determine the level of awareness and knowledge of medical practitioners regarding MM in Tshwane Municipality, Gauteng province, SA. This study will assist medical practitioners to have a high index of suspicion when patients present for the first time with symptoms, leading to early diagnosis thus preventing complications.

## Methods

### Study design and setting

A cross-sectional descriptive study was carried out among medical practitioners in three district, one regional and one central hospitals. The tertiary hospital was used as a pilot site, and the data obtained was included in the main data as there were no changes made. Communication was sent via emails and telephonically to four district and one regional hospitals. After engaging with management for all five hospitals for more than a year, permission was only obtained from four of them. Data were collected over 3 months from 04 March 2019 to 31 May 2019, and during the study period, the selected hospitals had 157 medical practitioners (Table 1).

**TABLE 1:** Summary of the number of doctors per hospital.

Hospitals	Number of doctors	Sample size proportional to size
A	40	19
B	60	28
C	27	13
D	10	5
E	20	9

**Total**	**157**	**74**

The bed capacity for the district and regional hospitals ranges from 50 to 414 beds. There are, on average, 53 clinics that are referring to these hospitals. The average distance from these hospitals to a higher level of care is approximately 30 km.^31^

### Study population, inclusion and exclusion criteria

The study population included all qualified medical practitioners such as interns, community service officers, medical officers, registrars and specialists in other disciplines working at the selected hospitals. The study excluded all clinical managers because the majority do administrative duties.

### Sample size and sampling procedures

A minimum sample size of 74 medical practitioners was required for this study, considering a study population of 157, a 95% confidence interval and a sampling error of 5%. The calculation of the sample size was performed in the Epi-Info program version 3.01 and allocated proportionally to the selected hospitals based on the number of medical practitioners (Table 1). The medical practitioners were recruited using a non-random convenience sample, and all (*N* = 157) received an invitation to participate in the study through their clinical managers, with a weekly follow-up reminder. The medical practitioners available during the facility visits were included in the study.

### Data collection

The self-administered questionnaire was used to collect the data. The researchers developed the questionnaire by reviewing relevant literature.^25,26,27,28^ The questionnaire has four parts. Section A is about respondents’ demographic data such as age, gender, rank, year in which the medical degree was completed, discipline and three yes and no questions on the education obtained on multiple myeloma. Section B and C consist of four (4) questions that assessed awareness and nine (9) questions for knowledge of MM. The answers to the questions were true or false and do not know, and the correct answers were coded as 1, incorrect and do not know as 0.

The score was calculated for each participant by summing up the points of all the questions and the score ranged from 0 to 4 for awareness and 0–9 for knowledge. Participants whose scores were 50% or more were considered to be aware and knowledgeable of MM practices. The questionnaire content validation and relevance were performed by a panel of independent consultants in the discipline of haematological pathology and piloted at DGMAH. Section D covered the respondents’ exposure and need for training.

### Data analysis

The data were entered into Microsoft Excel 2016 (Microsoft Corporation, Redmond, Washington, DC, United States) and analysed using SPSS^®^ statistical software (version 13.0 SPSS Inc, Chicago, Illinois) respectively. The percentages and numbers were used to present categorical data such as gender (i.e., male/female), years completed medical degree (i.e., ≤ 5, 6–10, 11–19 and 20+), rank (medical interns, community service officer (CSO), medical officer (MO), registrar, family physician, other speciality specified) and discipline, whereas median and interquartile ranges were used for the continuous variables (i.e., age, awareness and knowledge score for MM).

Logistic regression was used to determine associations between dependent variables (i.e., medical practitioners’ awareness and knowledge regarding MM) and independent variables (i.e., age, gender, years completed medical degree and rank). In a bivariate logistic regression analysis, a *p*-value of less than 0.05 was considered statistically significant.

### Ethical considerations

The study obtained ethical approval from Sefako Makgatho Health Sciences University Research Ethics Committee (Ref: SMUREC/M/178/2017). The permission to conduct the study was obtained from the Gauteng Provincial Department of Health and the superintendents of each hospital. All the participants completed the informed consent before completing the questionnaire and were assured of anonymity.

## Results

### Demographic characteristics

Seventy-four medical practitioners participated in this study (response rate: 100%). Their median age was 37 years, with an interquartile range (IQR) of 43–30 years. More than half of the doctors were medical officers aged < 40 years. Fifty-two percent of the doctors were ≤ 10 years of experience post-medical degree. Males and females were equally distributed (Table 2).

**TABLE 2:** Demographic characteristics of the respondents (*N* = 74).

Variable	*n*	%
**Age (years)**		
< 30	17	23
30–39	22	30
40–49	24	32
50+	8	11
Missing	3	4
**Gender**		
Male	36	49
Female	36	49
Missing	2	2
**Rank**		
Medical interns	9	12
CSO	3	4
MO	40	54
Registrar	11	15
Specialist	9	12
Missing	2	3
**Years completed a medical degree**		
≤ 5	25	34
6–10	13	18
> 10	30	40
Missing	6	8
**Discipline**		
Family Medicine	7	78
Obstetric and Gynaecology	1	11
Paediatric	1	11

CSO, community service officer; MO, Medical officer.

### Level of awareness and knowledge of multiple myeloma

**FIGURE 1:**
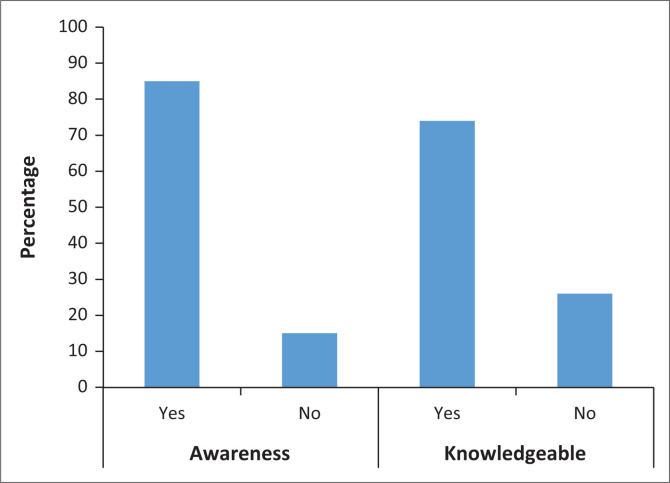
Level of awareness and knowledge about multiple myeloma.

The median score for awareness of MM was 4 (IQR: 4–3), and 85% of the respondents were aware of MM as a medical condition. The median score for knowledge of MM was 6 (IQR: 7–4). Seventy-four percent of the respondents were knowledgeable about MM presentation and diagnostic investigations (Figure 1).

Regarding individual items for awareness and knowledge of MM, the most frequent response was that MM is a malignant plasma cell disorder, not an infectious condition and can be diagnosed by markedly elevated erythrocyte sedimentation rate (ESR) and pathological fractures. The level of awareness and knowledge by rank is shown in Table 3.

**TABLE 3:** Awareness and knowledge of multiple myeloma by ranks.

Rank	*N*	Aware*		Knowledgeable**	
		*n*	%	*n*	%
Medical interns	9	9	100	4	44
CSO	3	3	100	3	100
MO	40	34	85	31	78
Registrar	11	9	82	9	82
Specialist	9	6	67	6	67

CSO, community service officer; MO, medical officer.

* , *p* = 0.496;

** , *p* = 0.324.

As shown in Table 4, male medical practitioners, ≥ 10 years of experience were more aware of MM when compared to other categories of doctors.

**TABLE 4:** Association between awareness of multiple myeloma and demographics.

Variable	Aware				Bivariate logistic regression		
	Yes		No		OR	95% CI	*p*
	*n*	%	*n*	%			
**Age (years)**							0.736
< 40	34	87	5	13	Ref	-	-
40+	27	84	5	16	0.8	0.2; 3.0	-
**Gender**							0.057
Female	28	78	8	22	Ref	-	-
Male	34	94	2	6	4.9	0.9; 24.7	-
**Rank**							
Specialist	6	67	3	33	Ref	-	-
MO	34	85	6	15	2.8	0.5; 14.5	0.212
Registrar	9	82	2	18	2.3	0.2; 17.8	0.442
CSO/Interns	12	100	0	0	1.0	0.3; 1.2	0.550
**Years since completing a medical degree**							0.164
≤ 10	34	90	4	10	Ref	-	-
> 10	23	77	7	23	0.4	0.1; 1.5	-

CSO, community service officer; MO, medical officer; CI, confidence interval.

Although male medical practitioner, medical officers, registrar and who had 10 or more years of experience were more knowledgeable than the other groups, the results were not statistically significant (*p* < 0.05; Table 5).

**TABLE 5:** Association between knowledge of multiple myeloma and demographics.

Variable	Knowledgeable				Bivariate logistic regression		
	Yes		No		OR	95% CI	*p*
	*n*	%	*n*	%			
**Age (years)**							0.543
< 40	28	72	11	28	Ref	-	-
40+	25	78	7	22	1.4	0.5; 4.2	-
**Gender**							0.789
Female	26	72	10	28	Ref	-	-
Male	27	75	9	25	1.2	0.4; 3.3	-
**Job category**							
Specialist	6	67	3	33	Ref	-	-
MO	31	78	9	22	1.7	0.4; 8.2	0.498
Registrar	9	82	2	18	2.3	0.3; 17.8	0.442
CSO/Interns	7	58	5	42	0.7	0.1; 4.2	0.698
**Years since completing a medical degree**							0.835
≤ 10	27	71	11	29	Ref	-	-
> 10	22	73	8	27	1.1	0.4; 3.3	-

CSO, community service officer; MO, medical officer; CI, confidence interval.

Regarding training on MM diagnosis, 69% of the participants said they received training about MM practices as undergraduate students, but few (8%) said they had attended an educational event on MM after qualifying (Table 6). Nearly all (98.7%) participants said they would benefit from an educational event and information brochure on MM.

**TABLE 6:** Education on multiple myeloma.

Variable	Yes		No	
	*n*	%	*n*	%
Received training about MM as an undergraduate student?	51	69.0	24	31.0
Ever attended an educational event on MM post-graduation?	6	8.0	68	92.0
Would you benefit from an educational event/information brochure on multiple myeloma?	73	98.7	1	1.3

MM, multiple myeloma.

## Discussion

This is the first study to investigate the level of awareness and knowledge of MM and associated factors among medical practitioners in public hospitals in SA. The findings show that more than two-thirds of the participants had a high level of awareness and knowledge of MM. Studies conducted in the United Kingdom (UK) found that under 60% of the general practitioners (GPs) or trainees (*n* = 58)^26^ and haematology healthcare professionals (*n* = 55),^25^ had knowledge and awareness, but these studies assessed the level of awareness and knowledge of MGUS. In Kenya^27^ and Nigeria,^28^ researchers reported very low awareness of the signs and symptoms of MM among doctors without conducting formal surveys. The reason for the higher level of awareness and knowledge in our study is not clear, but it could be that individual practitioners may have seen more MM cases throughout their careers given that MGUS is mainly diagnosed by pathologists when patients do not meet diagnostic criteria for MM.^11,12,13^

Interestingly, even though our findings revealed a high level of awareness and knowledge of MM as compared to UK, Kenya and Nigeria, nearly all (98.7%) of the medical practitioners in our study requested an educational brochure on MM practices. This is supported by the high number of MM patients referred to our tertiary setting with advanced stage of the disease, which could be related to the low index of suspicion of MM among medical practitioners.^13,21,22,23,24^ General practitioners and medical officers at district hospitals need to be aware of the disease as the majority of the patients with non-specific symptoms of MM get to be seen by them as their first point of call from the clinics. This will save many patients from developing complications such as pathological fractures.

Concerning the demographics, more than half (54%) of respondents were MOs, which shows the significant role played by these health workers in the diagnostic referral pathways of MM. This concurs with the findings of previous studies, which show that the majority of MM patients initially consulted a GP outside the haematology unit.^23,32,33,34^ Therefore, appropriate awareness and knowledge of MM among GPs are essential for early diagnosis and referral of MM cases. In our study, slightly one-third (34%) of the participants had completed their medical degree within the last 5 years, which is lower than 43.1% reported in a UK study that showed lower awareness and knowledge of MGUS among GPs and/or primary care physicians.^26^

Elliss-Brookes and colleagues in their UK study found that many MM cases (37%) were diagnosed within the emergency department, 13% in other outpatient departments and 27% were GP referrals.^35^ In contrast, a retrospective study carried out among 582 patients at the Steve Biko Academic Hospital in SA found that the majority of the MM patients were commonly diagnosed in orthopaedic and internal medicine.^13^ Interestingly, in the present study, the majority (63%) of the medical practitioners were stationed in family medicine, internal medicine, general surgery, orthopaedic and emergency department. Thus, the medical practitioners in these disciplines are more likely to have seen more MM cases, which supports the high level of awareness and knowledge of MM observed in this study. Our findings showed that male practitioners were five times more likely to be aware of the MM than females, and this could be because males (61%) made up the majority of the GPs compared to 39% of females, of which GPs are the most common practitioners patients initially consult.^17,36,37^ Our finding also found that participants with six or more years post-medical degrees were aware of MM, which shows that most of the respondents in our study were experienced.

The Tackling Early Morbidity and Mortality in Multiple Myeloma (TEAMM) trial undertaken in the UK evaluated the routes to diagnosis in patients with myeloma and the relationship between diagnostic pathways, time to diagnosis and disease severity among 915 patients. This UK study found that 51% of the patients were diagnosed by direct referral from primary care to haematology, while 29% and 20% were diagnosed and referred via acute services and other specialities, respectively.^38^ The TEAMM trial also noted that patients diagnosed via other secondary care specialities significantly had a longer diagnostic interval^38^ and most of these patients were found to experience the highest frequency of complications.^23,38^ Although our findings showed a good level of awareness and knowledge about MM, the late presentation of patients to a healthcare facility^13,19,20^ because of a low level of suspicion by clinicians,^13^ the inadequacy of diagnostic infrastructure in the facilities,^20^ diagnostic and referral delays of MM among GPs^24^ remains a challenge in sub-Saharan African countries. Therefore, there is a need to raise awareness of this condition among the general public, address diagnostic infrastructural deficiencies in healthcare facilities and improve referral pathways from primary care physicians to a haematologist.

This study has several limitations. It is a single time point study, with a small sample size and involved medical practitioners in one of the three municipalities in Gauteng Province; therefore, the results cannot be generalised to all medical practitioners working in hospitals in the province. The limitation of this study is also acknowledged for not assessing the medical practitioners’ practices of MM, which could assist in determining the reasons for the low suspicion index of MM among clinicians resulting in patients’ initial symptoms being ignored or missed.

The other limitation of the study is that the level of awareness and knowledge was not shown at different levels of care (district, regional and central hospitals). This finding would give an idea if where the patients present first there was a lack of awareness or knowledge or vice versa. Despite these limitations, this study established a high awareness and knowledge of MM in public hospitals in the Tshwane region. Factors associated with awareness include gender and years of completing a medical degree.

## Conclusion

In conclusion, our findings have highlighted a higher proportion of the participants had a high level of awareness and knowledge of MM. Despite this, MM patients referred to our tertiary setting were found in the advanced stage of the disease. Given the challenges in suspecting MM in patients in a primary care setting and the worse disease-free survival rate with debilitating complications seen in our patients, our study recommends continuous training of medical practitioners through continued professional development (CPD) meetings at the district hospitals, to increase their index of suspicion for MM.

In addition, medical practitioners from referring institutions without comprehensive cancer treatment facilities should be encouraged to timely refer MM patients to haematological services on time. Further studies with a larger sample are required to assess the level and identify factors associated with awareness, knowledge and practices of MM among medical practitioners. Moreover, other studies are required to assess healthcare professionals that work at non-specialised units such as casualty as well as primary care nurses as most primary health care clinics in South Africa are nurse-driven.
